# Supplementary material for: Liebenberg syndrome severity arises from variations in Pitx1 locus topology and proportion of ectopically transcribing cells
Source: Nat Commun. 2025 Jul 9;16:6321. doi: 10.1038/s41467-025-61615-2 (PMC12241559; doi:10.1038/s41467-025-61615-2)
Supplement: Supplementary file 1 — Supplementary Information [file 41467_2025_61615_MOESM1_ESM.pdf]

Supplementary information to

Liebenberg syndrome severity arises from variations in *Pitx1* locus topology and proportion of ectopically transcribing cells

Author list

Olimpia Bompadre[1,2], Raquel Rouco[1,2], Fabrice Darbellay[1,2], Antonella Rauseo[1,2], Fanny Guerard-Millet[3,4], Claudia Gentile[3, 5, 6], Marie Kmita[3, 4, 5] and Guillaume Andrey[1,2]

Affiliations

[1]Department of Genetic Medicine and Development, Faculty of Medicine, University of Geneva, Geneva, Switzerland
[2]Institute of Genetics and Genomics in Geneva (iGE3), University of Geneva, Geneva, Switzerland
[3]Genetics and Development Research Unit, Institut de Recherches Cliniques de Montréal, Montréal, QC H2W 1R7, Canada
[4]Department of Medicine, Université de Montréal, Montréal, QC H3T 1J4, Canada
[5]Department of Medicine, Division of Experimental Medicine, McGill University, Montréal, QC H4A 3J1, Canada
[6]Current Address: Dana-Farber Cancer Institute and Harvard Medical School, 450 Brookline Avenue, Boston, MA, 02215, USA
Correspondence: guillaume.andrey@unige.ch

Supplementary Figures

Supplementary Figure 1

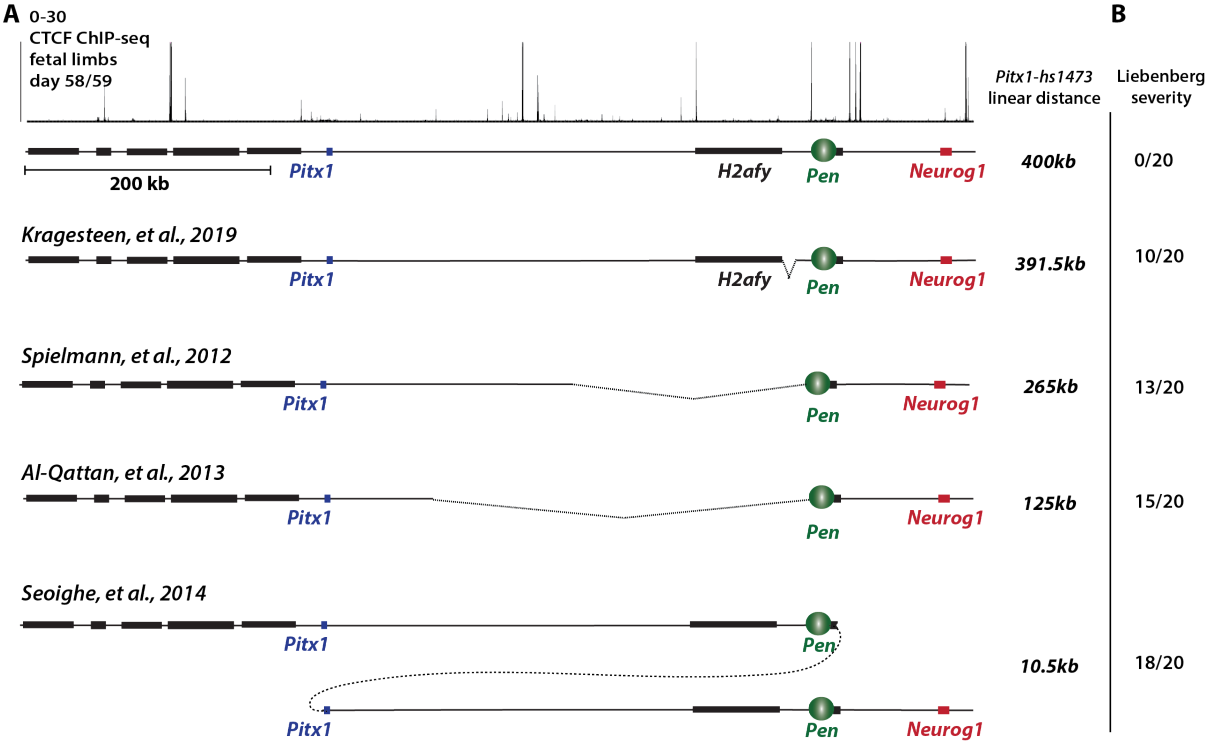

Supplementary Figure 1: *We categorised the phenotypic description of Liebenberg anatomical features assigning a score based on the severity of the malformation: 0= not mentioned, 1= partially malformed and 2= severely malformed, taking also into account the physician's own comments on the severity of the cases (**Supplementary Table 1**). From this emerged that the more the distance between Pitx1 and Pen was reduced, the more severe was the manifestation of the condition (**Figure S2B**).*

Supplementary Figure 2

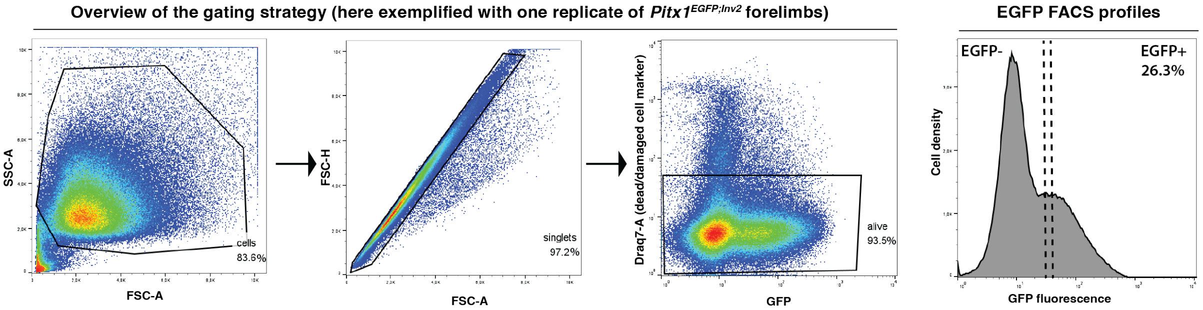

Supplementary Figure 2: *Graphical depiction of the gating strategy used to separate EGFP+ and EGFP- cell groups (here showcased with one replicate of Pitx1$^{EGFP;Inv2}$ forelimbs).*

Supplementary Figure 3

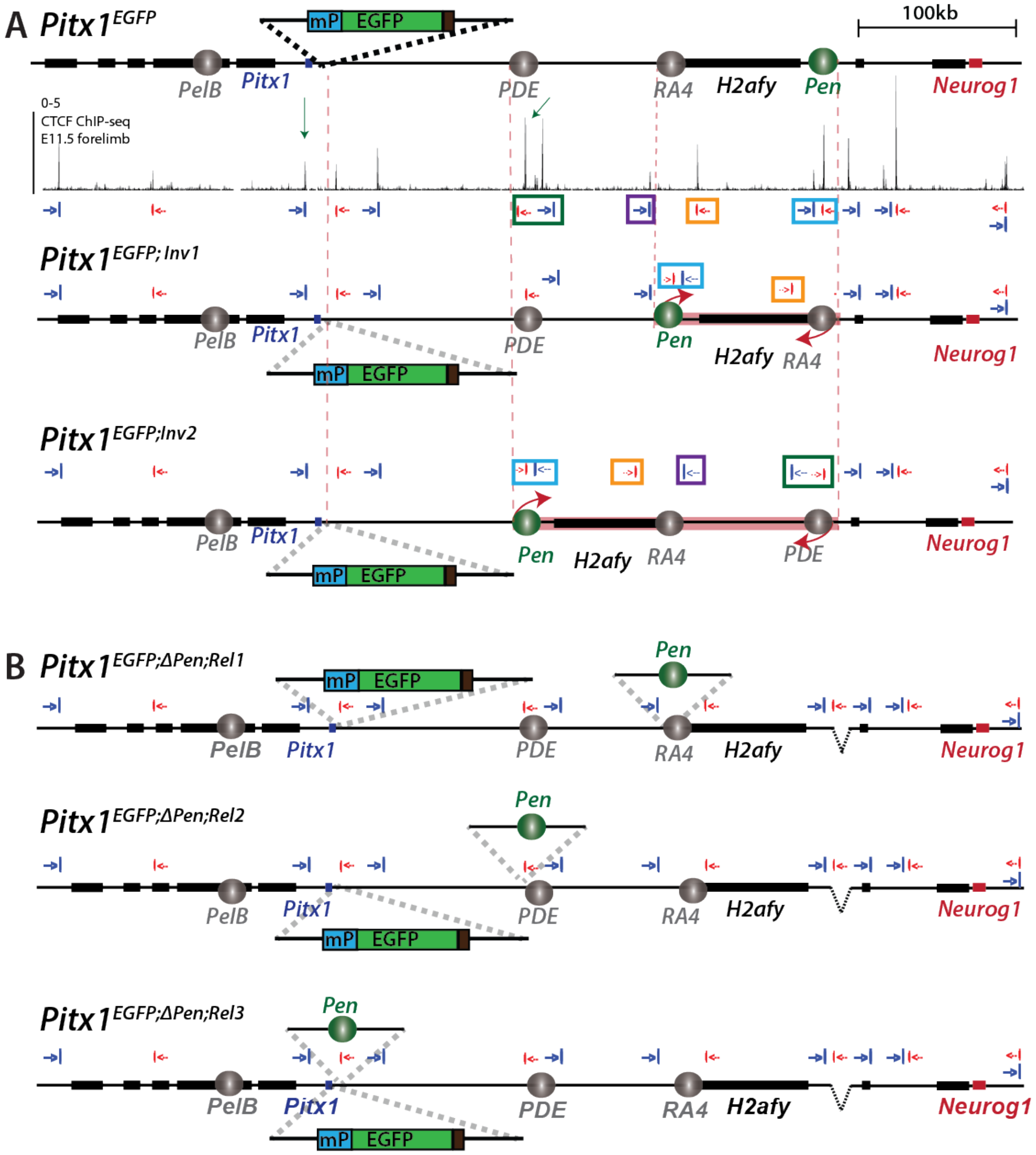

Supplementary Figure 3: A. *ChIP-seq of CTCF binding at the Pitx1 locus shows several binding sites through the locus, in coloured boxes we highlight CTCF binding sites whose directionality is disrupted by the inversions Pitx1$^{EGFP;Inv1}$ and Pitx1$^{EGFP;Inv2}$, these CTCF sites are represented in the SVs at their new location and directionality. Red and blue arrows show CTCF binding site orientation, red dotted lines show the breakpoints of the SVs, green arrows on the CTCF ChIP-seq track indicates the two CTCF sites that form a stable loop between Pitx1 and PDE (Andrey, et al, 2017).* ***B.*** *A representation of the relocation of Pen approach (Pitx1$^{EGFP;\Delta Pen;Rel1}$, Pitx1$^{EGFP;\Delta Pen;Rel2}$* and *Pitx1$^{EGFP;\Delta Pen;Rel3}$) in a Pitx1$^{EGFP;\Delta Pen}$ background, where CTCF site orientation and position in the locus is not disrupted.*

Supplementary Figure 4

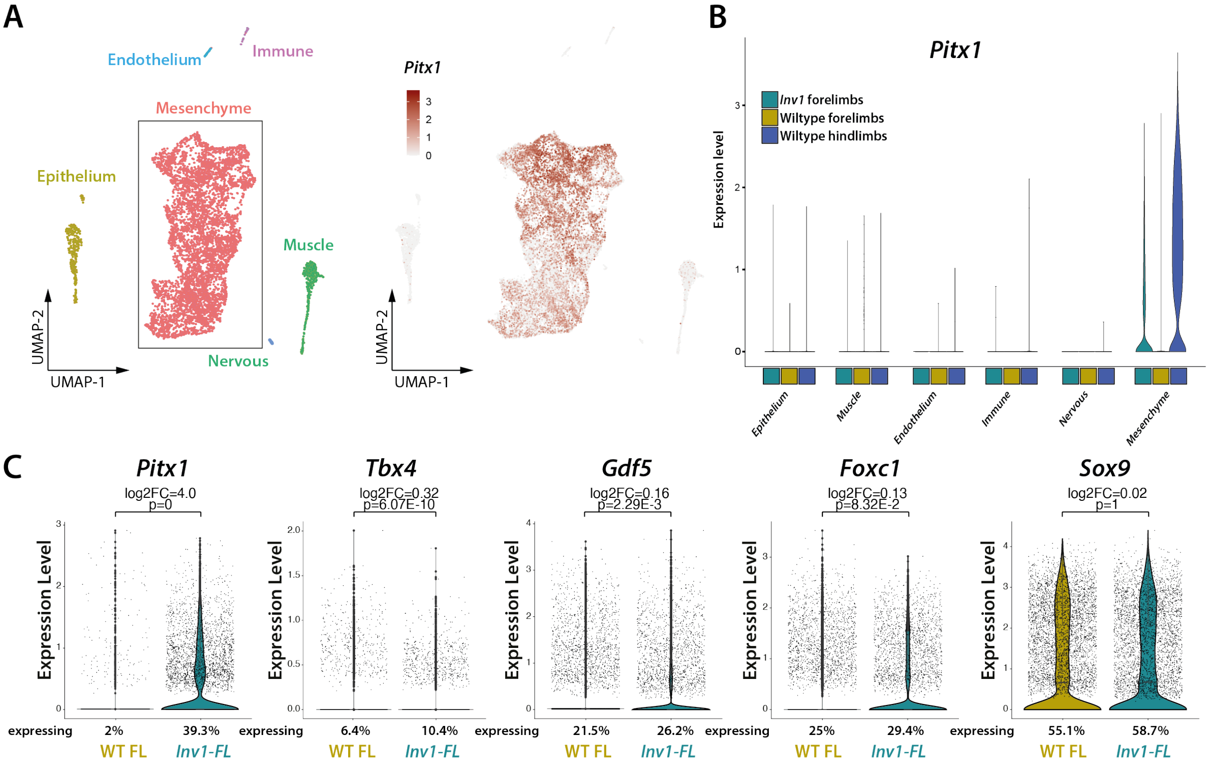

Supplementary Figure 4:* *A. *Left: UMAP of cell clusters present in wildtype fore- and hindlimbs and* $Pitx1^{Inv1/+}$ *hindlimbs. The black square indicates the mesenchymal cells reclustered in Figure 4A. Right: UMAP representation of Pitx1 expression across cell types.* ***B.*** *violin plots show expression levels of Pitx1 divided by limb identity and cell cluster, Pitx1 expression is restricted to mesenchymal cells and it is absent in wildtype forelimbs and present in wildtype hindlimbs* and $Pitx1^{Inv1/+}$ *forelimbs (Inv1 FL).* ***C.*** *Violin plots of Pitx1, Tbx4, Gdf5, Foxc1 and Sox9 expression in wildtype and Inv1 forelimb mesenchymal cells. Black dots represent single cell expression values. The numbers below the graph indicate the proportion of expressing cells. "log2FC" indicate Log2 fold change and "p" correspond to adjusted p-value, calculated using the Wilcoxon Rank Sum test via the FindMarkers function from the Seurat R package. Of note, the 39.3% of Pitx1-expressing cells differs from the 34% mentioned in the main text, as this percentage is calculated specifically within the mesenchymal subpopulation rather than across all limb cell clusters.*

Supplementary Figure 5

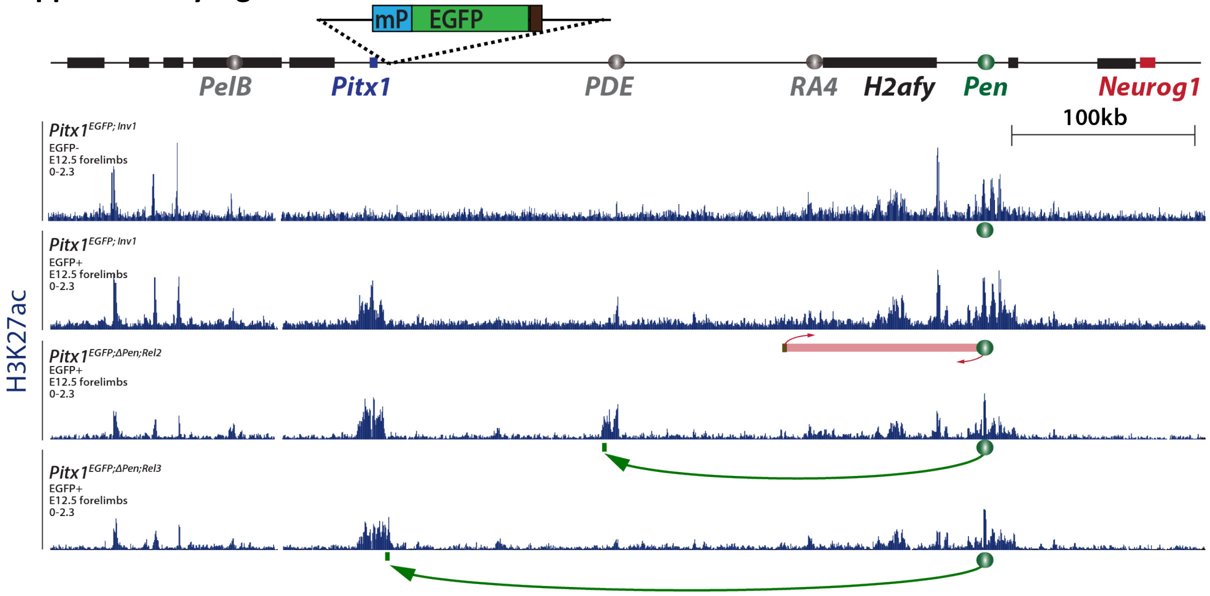

Supplementary Figure 5: *A. H3K27ac ChIP-seq GFP- cells from Pitx1$^{EGFP;Inv1}$ as well as EGFP+ cells from Pitx1$^{EGFP;Inv1}$, Pitx1$^{EGFP;\Delta Pen;Rel2}$ and Pitx1$^{EGFP;\Delta Pen;Rel3}$ E12.5 forelimbs. Reads were mapped to a wildtype mm39 mouse reference genome. Note the real position of the Pen enhancers indicated by a green box within the inverted red box for the inversion, and by arrows for the two relocations* (n=1).

Supplementary Figure 6

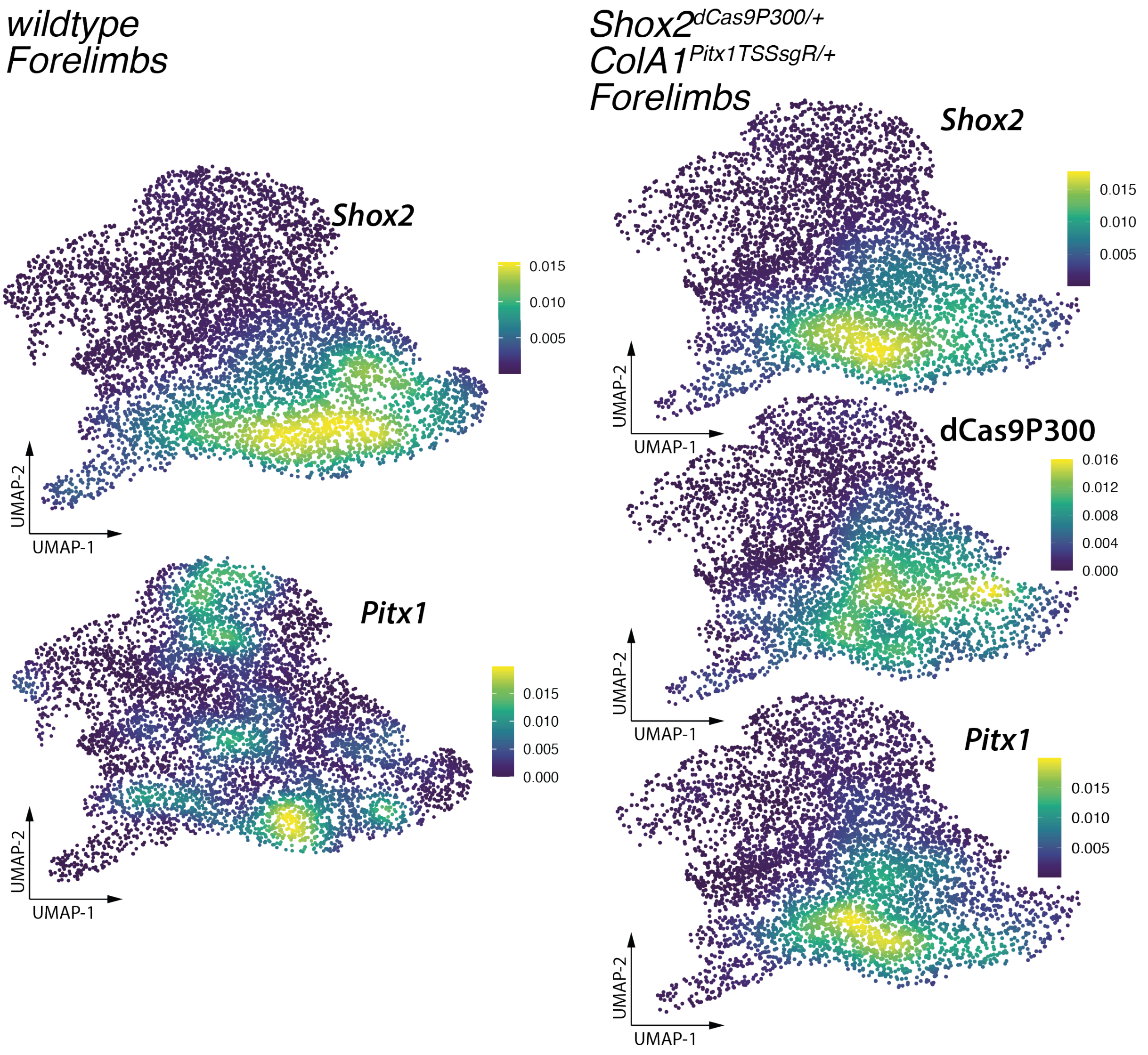

***Supplementary Figure 6**: Density plots of scRNA-seq data from wildtype forelimbs and Shox2$^{dCas9P300/+}$;ColA1$^{TSSsgR/+}$ forelimbs shows Shox2, Pitx1 and dCas9P300 expressing cells and co-localisation.*

Supplementary Figure 7

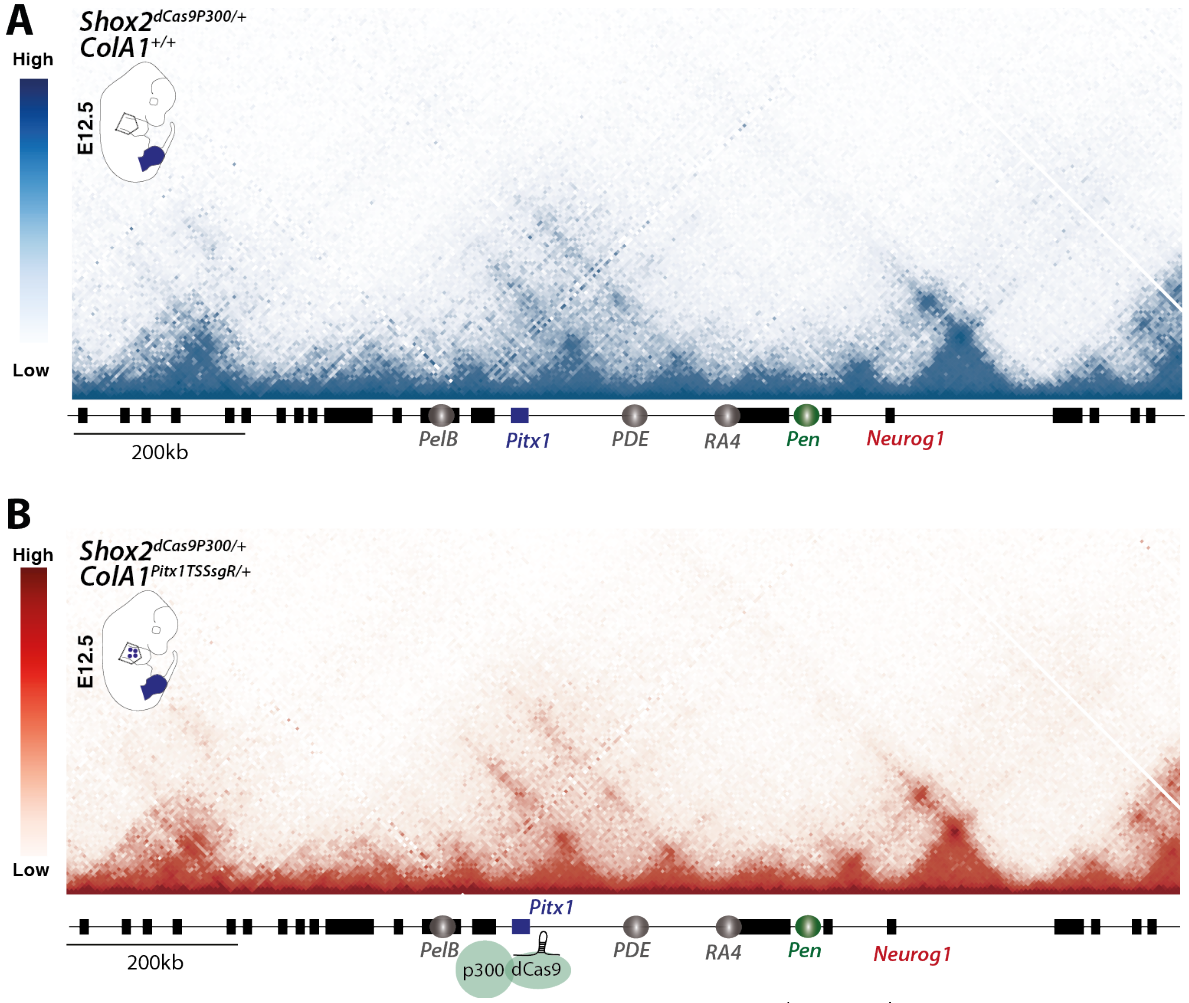

Supplementary Figure 7: *C-HiC maps of (**A**) Shox2$^{dCas9P300/+}$;ColA1$^{+/+}$ (no sgRNA) and (**B**) Shox2$^{dCas9P300/+}$;ColA1$^{TSSsgR/+}$ (TSS sgRNA) E12.5 proximal forelimbs at the Pitx1 locus. Darker red or blue bins indicate stronger interaction frequencies as shown on the scale bars* (n=1).

Supplementary Figure 8

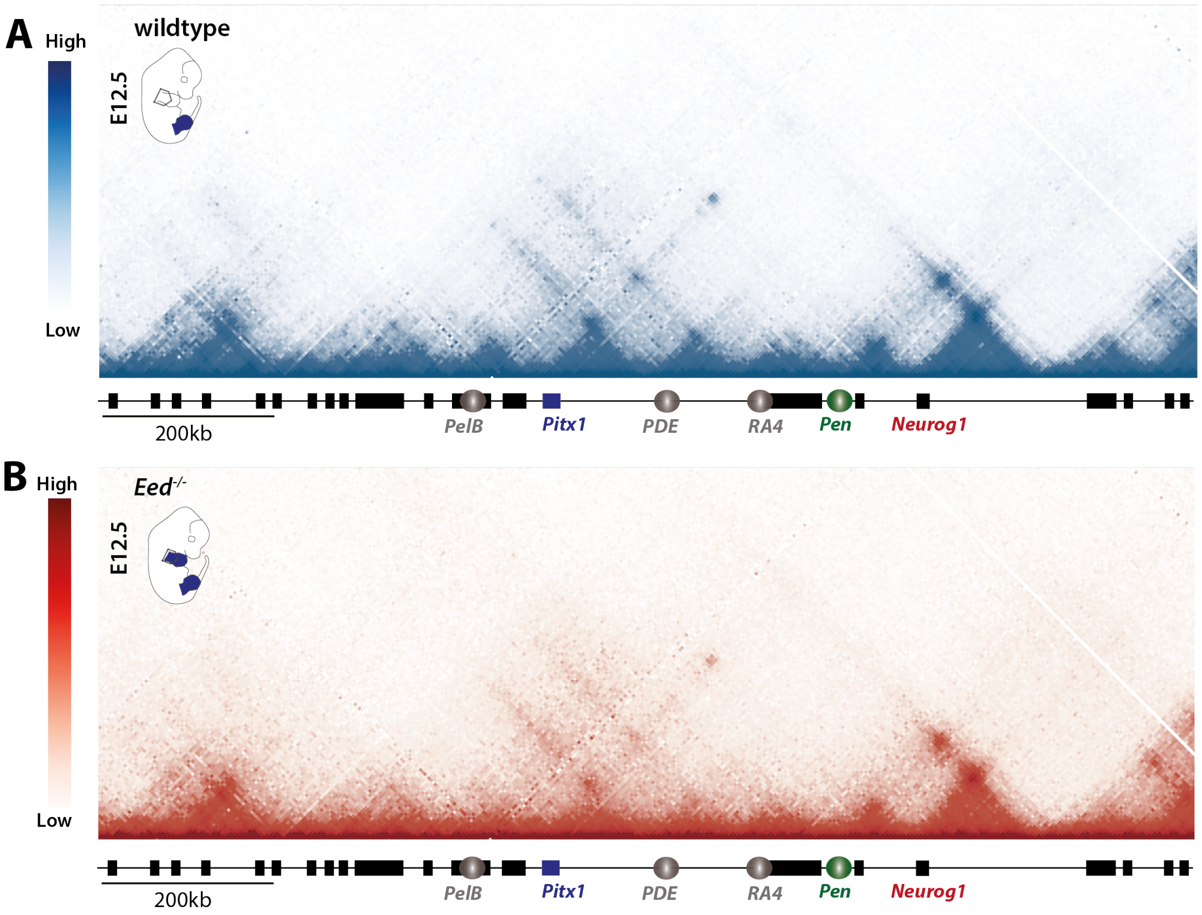

Supplementary Figure 8: *C-HiC maps of (**A**) wildtype and (**B**) Prx1-Cre;$Eed^{flox/-}$ ($Eed^{-/-}$) E12.5 proximal forelimbs at the Pitx1 locus. Darker red and blue bins indicate stronger interaction frequencies as shown on the scale bars* (n=1).

Supplementary Figure 9

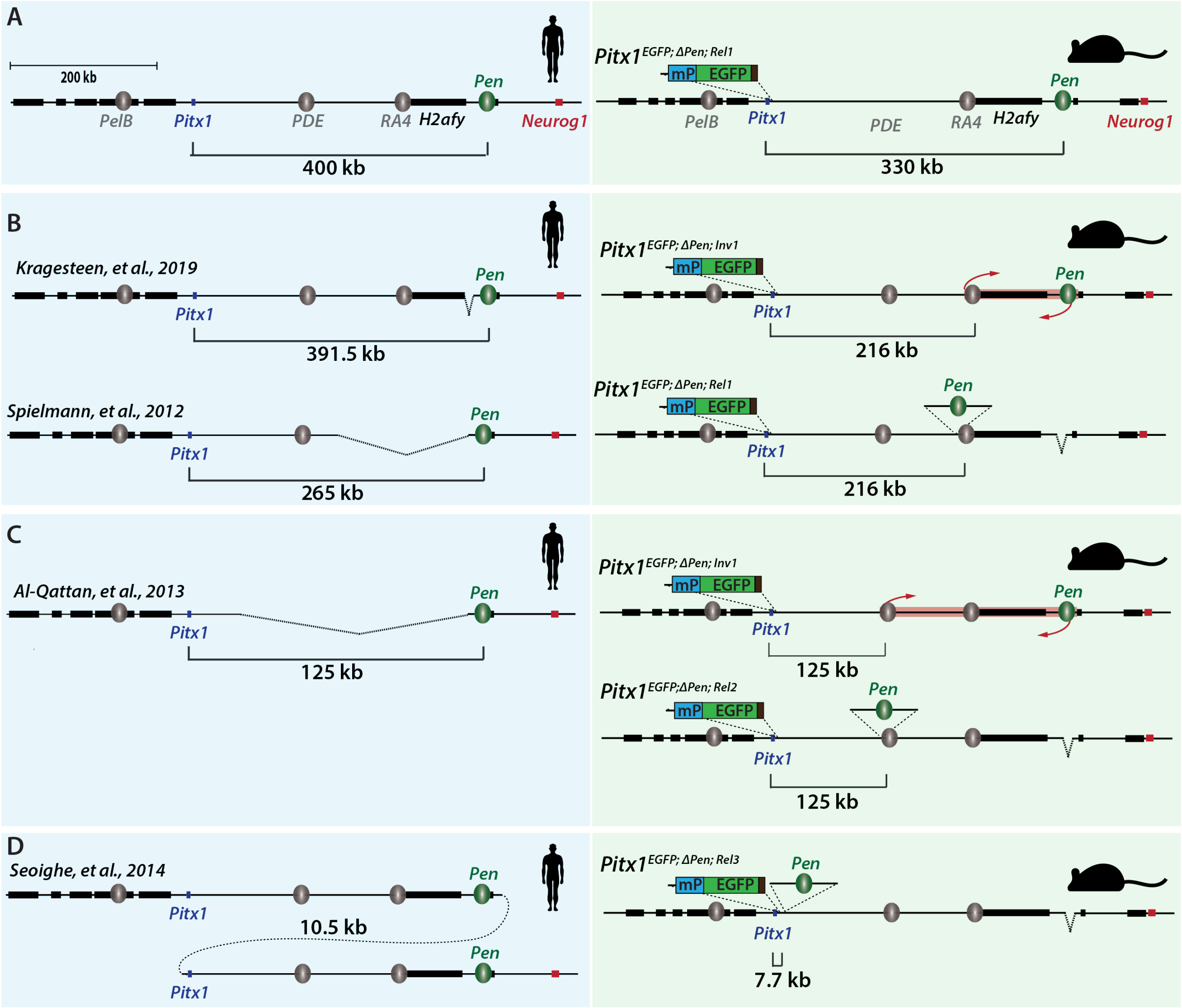

Supplementary Figure 9: *Comparison of human patient and mouse re-arrangements. (**A**) The wildtype Pitx1 locus with a Pitx1-Pen genomic distance of 400kb (human) and 330kb (mice). (**B**) Small re-arrangements with Pitx1-Pen distance reduced by 10-100kb. (**C**) Intermediate re-arrangements, with a Pitx1-Pen distance of 125kb. (**D**) Large re-arrangements with a Pitx1-Pen distance < 15kb.*
