# Supplementary material for: Liebenberg syndrome severity arises from variations in Pitx1 locus topology and proportion of ectopically transcribing cells
Source: Nat Commun. 2025 Jul 9;16:6321. doi: 10.1038/s41467-025-61615-2 (PMC12241559; doi:10.1038/s41467-025-61615-2)
Supplement: Supplementary file 2 — Description of Additional Supplementary Files [file 41467_2025_61615_MOESM2_ESM.pdf]

**Description of Additional Supplementary Files**

**File name:** Supplementary Data 1

**Description:** Scoring of reported cases of Liebenberg syndrome based on anatomical features

**File name:** Supplementary Data 2

**Description:** Combined results of the differential expression analyses presented in Figure 1 and Figure 2. Normalized FPKMs for all datasets are provided, followed by DESeq2 pairwise comparisons (*padj* is the FDR-corrected, two-tailed p-value using the Benjamini-Hochberg method). "GFPp" indicated FACS sorted GFP+ cell populations, "HLWT" indicated E12.5 $Pitx1^{EGFP/EGFP}$ hindlimbs, "FLWT" indicated E12.5 $Pitx1^{EGFP/EGFP}$ forelimbs. *Inv1*, *Inv2*, *Rel1*, *Rel2* and *Rel3* correspond to $Pitx1^{EGFP;Inv1/EGFP;+}$, $Pitx1^{EGFP;Inv2/EGFP;+}$, $Pitx1^{EGFP;\Delta Pen;Rel1/EGFP;\Delta Pen;+}$, $Pitx1^{EGFP;\Delta Pen;Rel2/EGFP;\Delta Pen;+}$, $Pitx1^{EGFP;\Delta Pen;Rel3/EGFP;\Delta Pen;+}$ E12.5 forelimbs, respectively.

**File name:** Supplementary Data 3

**Description:** Phenotypic scoring of E18.5 fetuses for skeletal defects. The penetrance of phenotypes is also reported.

**File name:** Supplementary Data 4

**Description:** scRNA-seq marker genes for all E12.5 limb clusters, as well as for re-clustered mesenchymal cells. Two-sided Wilcoxon Rank Sum test, p-value adjustment is performed using Bonferroni correction based on the total number of genes in the dataset.

**File name:** Supplementary Data 5

**Description:** Combined results of differential expression analysis of bulk E12.5 forelimbs. "FLWT" indicated E12.5 $Pitx1^{EGFP/EGFP}$ forelimbs, *Rel2* and *Rel3* correspond to $Pitx1^{EGFP;\Delta Pen;Rel2/EGFP;\Delta Pen;+}$, $Pitx1^{EGFP;\Delta Pen;Rel3/EGFP;\Delta Pen;+}$ forelimbs, respectively. Normalized FPKMs for all datasets are provided, followed by DESeq2 pairwise comparisons (*padj* is the FDR-corrected, two-tailed p-value using the Benjamini-Hochberg method).

**File name:** Supplementary Data 6

**Description:** Differential expression analysis of E12.5 *Shox2*$^{dCas9P300/+}$*;ColA1*$^{+/+}$ (dCas9P300-no-sgRNA) and *Shox2*$^{dCas9P300/+}$*;ColA1*$^{TSSsgR/+}$ (dCas9P300-TSS) forelimbs. FPKM and normalized counts are provided. Pairwise DESeq2 was performed for differential analysis (*padj* is the FDR-corrected, two-tailed p-value using the Benjamini-Hochberg method).

**File name:** Supplementary Data 7

**Description:** Differential expression analysis of E12.5 *Prx1-Cre;Eed*$^{flox/-}$ (*Eed*) and wildtype (WT) forelimbs. FPKM and normalized counts are provided. Pairwise DESeq2 was performed for differential analysis (*padj* is the FDR-corrected, two-tailed p-value using the Benjamini-Hochberg method).

**File name:** Supplementary Data 8

**Description:** sgRNAs for CRISPR/Cas9, sgRNAs for dCas9-P300 and primers.
