# Supplementary material for: Liebenberg syndrome severity arises from variations in Pitx1 locus topology and proportion of ectopically transcribing cells
Source: Nat Commun. 2025 Jul 9;16:6321. doi: 10.1038/s41467-025-61615-2 (PMC12241559; doi:10.1038/s41467-025-61615-2)
Supplement: Supplementary file 11 — Reporting Summary [file 41467_2025_61615_MOESM11_ESM.pdf]

nature portfolio

Corresponding author(s): Guillaume Andrey

Last updated by author(s): Apr 29, 2025

Reporting Summary

Nature Portfolio wishes to improve the reproducibility of the work that we publish. This form provides structure for consistency and transparency in reporting. For further information on Nature Portfolio policies, see our Editorial Policies and the Editorial Policy Checklist.

Statistics

For all statistical analyses, confirm that the following items are present in the figure legend, table legend, main text, or Methods section.

n/a	Confirmed	
☐	☒	The exact sample size (*n*) for each experimental group/condition, given as a discrete number and unit of measurement
☐	☒	A statement on whether measurements were taken from distinct samples or whether the same sample was measured repeatedly
☐	☒	The statistical test(s) used AND whether they are one- or two-sided<br>*Only common tests should be described solely by name; describe more complex techniques in the Methods section.*
☒	☐	A description of all covariates tested
☐	☒	A description of any assumptions or corrections, such as tests of normality and adjustment for multiple comparisons
☐	☒	A full description of the statistical parameters including central tendency (e.g. means) or other basic estimates (e.g. regression coefficient) AND variation (e.g. standard deviation) or associated estimates of uncertainty (e.g. confidence intervals)
☐	☒	For null hypothesis testing, the test statistic (e.g. *F*, *t*, *r*) with confidence intervals, effect sizes, degrees of freedom and *P* value noted<br>*Give P values as exact values whenever suitable.*
☒	☐	For Bayesian analysis, information on the choice of priors and Markov chain Monte Carlo settings
☒	☐	For hierarchical and complex designs, identification of the appropriate level for tests and full reporting of outcomes
☒	☐	Estimates of effect sizes (e.g. Cohen's *d*, Pearson's *r*), indicating how they were calculated

Our web collection on statistics for biologists contains articles on many of the points above.

Software and code

Policy information about availability of computer code

Data collection	No commercial, open source and custom code was used to collect the data in this study.
Data analysis	1- RNA-seqThe code is available on https://github.com/bompadreolimpia/Bompadre_etal_2024.<br>RNA-seq reads were processed using CutAdapt v1.18 to trim low-quality bases and NextSeq sequencing adapters (-a CTGTCTCTTATACACATCTCCGAGCCCACGAGAC, quality cutoff -q30 and minimum length required -m15). Unstranded reads were mapped to the relevant GRCm39/mm39 custom genome filtered GTFs (see Custom Genomes section below) with using the STAR 2.7.2b mapper with settings allowing for accurate gene quantification (--outSAMstrandField intronMotif--sjdbOverhang '99' -- sjdbGTFfile $gtfFile--quantMode GeneCounts--outFilterType BySJout--outFilterMultimapNmax 20 -- outFilterMismatchNmax 999--outFilterMismatchNoverReadLmax 0.04-- alignIntronMin 20 -- alignIntronMax 1000000--alignMatesGapMax 1000000--alignSJoverhangMin 8 -- alignSJDBoverhangMin 1). Output BigWig files were displayed on the UCSC genome browser. Counts were compiled from STAR counts using R 3.6.2, and FPKM were computed through Cufflinks 2.2.1 using the filtered GTFs created for this study (--max-bundle-length 10000000 -- max-bundle-frags 100000000 -- multi-read-correct--library-type "fr-firststrand" --no-effective-length-correction -M MTmouse.gtf). The code is available on https://github.com/bompadreolimpia/Bompadre_etal_2024. Normalized FPKM values were calculated by first determining coefficients extrapolated from a set of 1,000 housekeeping genes known for their stable expression as defined from the comparison of a series of RNA-seq (Brawand et al., 2011). The coefficients obtained were then applied to adjust the respective FPKM values. Differential expression analysis utilized the DEseq2 R package (version 1.38.3), with the Wald test for comparisons across samples and multiple test correction using the FDR/Benjamini-Hochberg test. Each analysis included two biological replicates per condition. Fold-enrichment of Pitx1 and was calculated using DEseq2's normalization by size factor. Count normalisation and differential expression analysis was done following published pipelines (https://github.com/lldelisle/rnaseq_rscripts).<br><br>2- Custom Genomes.

For RNA-seq analysis, custom mm39 genomes were generated using STAR 2.7.2b, incorporating an additional chromosome to accommodate the custom sequences of EGFP and SV40pA polyA tail. The gft file was modified to specify these sequences as coding genes and exons. Cell Ranger 6.1.2 was utilized to create the mm39_dCas9P300 custom genome used for RNA-seq and single-cell RNA-seq analysis, by adding an extra dCas9P300 -containing chromosome and customizing the reference gtf file. The code is available on https://github.com/bompadreolimpia/Bompadre_etal_2024.

3- ChIP-seq
Reads from ChIP-seq data generated for this study were pre-processed withCutAdapt v1.18 to trim low quality bases and TruSeq adapters (-a GATCGGAAGAGCACACGTCTGAACTCCAGTCAC, -q30 and -m15). Reads were then mapped to the reference GRCm39/mm39 genome using Bowtie2 2.3.5.1 with default settings. Only reads with mapping quality score (MAPQ) of 30 or above were retained by filtering with SAMtools v1.10. For coverage and peak analysis reads were extended by 200 bp and processed with MACS2 v2.2.7.1 (--broad --nolambda --broad-cutoff 0.05 --nomodel --gsize mm --extsize 200 -B 2). Coverage normalization was performed by MACS2, normalized by the number of million tags used by MACS2. BedGraphToBigWig v4 was used to convert files into BigWig format for visualization in the UCSC browser. Datasets of CTCF ChIP-seq of mouse embryonic E11.5 forelimb (Andrey et al., 2017) and human fetal limbs day 58/59 (ENCODE, annotation file set ENCSR191WSJ) were visualized in the UCSC browser. The code is available on https://github.com/bompadreolimpia/Bompadre_etal_2024.

4- Capture-HiC
Capture-HiC data analysis followed previous descriptions (Rouco et al., 2021). Briefly, reads were mapped against the reference NCBI37/mm9 genome using Bowtie2 v2.3.4.2. Filtering, de-duplication, and processing of valid pairs were done with HiCUP v0.6.1 with default parameters for configuration file, but adding Nofill: 1 as an additional parameter. Filtered di-tags were then processed with Juicer Tools v1.9.9. Binned contact maps were produced with MAPQ ≥30 valid and unique read pairs and maps were normalised using Knights and Ruiz matrix balancing and exported at 5kb resolution. Subtraction maps of the KR normalised maps were scaled together across their subdiagonals. All maps were visualised as heatmaps where values above the 99th percentile were truncated for visualisation purposes. The code is available on https://github.com/bompadreolimpia/Bompadre_etal_2024.

5- scRNA-seq.
Sequenced reads were mapped to the custom genome mm39_dCas9P300 and corresponding GTF file using the 10X Genomics Cell Ranger 6.1.2 software. Data filtering (nFeature_RNA > 200 & nFeature_RNA < 5000 & percent.mt < 5 & nCount_RNA > 1000 & nCount_RNA < 26000), quality control, normalization, scaling, dimensional reduction, and doublet identification were performed using Seurat 4.3.0 and DoubletFinder 2.0.3. Cells were further filtered to exclude blood cells present in our dataset (percent.mt > 1 & percent.mt < 5).
Merging and Normalization. Following individual dataset filtering and normalization, the two wildtype forelimb replicates and the two Pitx1Inv1+/- forelimb replicates were merged as single Seurat objects. To account for potential variance due to cell-cycle variations, cell cycle regression was implemented using the CellCycleScoring method with a predetermined list of marker genes (Tirosh et al., 2016). The dataset underwent additional normalization through SCTransform with standard parameters, incorporating the scored cell-cycle and the dCas9P300 feature as regressed variables (Hafemeister et al., 2019).
Clustering of Whole Limbs and Mesenchyme. The cells were clustered after cell cycle and dCas9P300 regression using the SCTransform Seurat package. For clustering, PCA (50 npcs) and UMAP (50 dims) were utilized, and the closest neighbors of each cell were calculated. The Seurat FindClusters function was employed with a resolution of 0.1, defining 9 clusters. Cluster identification was performed with the FindMarkers function, enabling the selection of differently expressed gene markers among clusters (ident.1, only.pos=TRUE). Three mesenchymal cell clusters and two epithelial cell clusters were merged, with 6 final clusters remaining, where the FindMarkers function was re-run.
Given the exclusive expression of Pitx1 and Shox2 in the mesenchymal cells of the limb, downstream analysis focused on these populations. The 3 mesenchymal cell populations were merged and reclustered. PCA of 20 npcs and UMAP of 20 dims were applied, and closest neighbours were calculated for each cell. Using Seurat FindClusters, 10 clusters were defined with a resolution of 0.3. FindMarkers was then run for each cluster, selecting gene markers (ident.1, only.pos=TRUE). Two Irregular Connective Tissue clusters were merged and FindMarkers was re-run on the final 9 clusters. UMAP density plots were obtained using the R package Nebulosa v1.8.0 and scTransform v0.4.1. The code is available on https://github.com/bompadreolimpia/Bompadre_etal_2024.
Expression correlation. To calculate the correlation of expression of two genes in a sample from single-cell-RNAseq data we employed baredSC v2.0.0 (Lopez-Delisle, et al, 2022). Here, the confidence interval of correlation is given as a percentage and the p-value, where p is the probability for the correlation coefficient to be negative, is the mean probability with the estimated standard deviation of this mean probability. The code is available on https://github.com/bompadreolimpia/Bompadre_etal_2024.

For manuscripts utilizing custom algorithms or software that are central to the research but not yet described in published literature, software must be made available to editors and reviewers. We strongly encourage code deposition in a community repository (e.g. GitHub). See the Nature Portfolio guidelines for submitting code & software for further information.

Data

Policy information about availability of data

All manuscripts must include a data availability statement. This statement should provide the following information, where applicable:

- Accession codes, unique identifiers, or web links for publicly available datasets
- A description of any restrictions on data availability
- For clinical datasets or third party data, please ensure that the statement adheres to our policy

Sequencing data are available in the GEO repository under the accession number GSE259212 [https://www.ncbi.nlm.nih.gov/geo/query/acc.cgi?acc=GSE259212].

Research involving human participants, their data, or biological material

Policy information about studies with human participants or human data. See also policy information about sex, gender (identity/presentation), and sexual orientation and race, ethnicity and racism.

Reporting on sex and gender	N/A
Reporting on race, ethnicity, or other socially relevant groupings	N/A
Population characteristics	N/A
Recruitment	N/A
Ethics oversight	N/A

Note that full information on the approval of the study protocol must also be provided in the manuscript.

Field-specific reporting

Please select the one below that is the best fit for your research. If you are not sure, read the appropriate sections before making your selection.

- [x] Life sciences
- [ ] Behavioural & social sciences
- [ ] Ecological, evolutionary & environmental sciences

For a reference copy of the document with all sections, see nature.com/documents/nr-reporting-summary-flat.pdf

Life sciences study design

All studies must disclose on these points even when the disclosure is negative.

Sample size	For scRNA-seq, sample size is a singlicate of 4 micro-dissected limb pairs, 7x104 cells were prepared for sequencing. For flow cytometry experiments, at least 4x104 cells were recorded by BioRad S3 per measurement. For bulk limb RNA-seq sample size was obtained from 2 biological replicates of 1-2 limbs. For FACS-sorted backed experiments, the minimum number of cells for any of the experimental procedures was determined by the type of experiment, i.e. RNA-seq 2.5x105 cells, ChIP-seq 5x105 cells, C-HiC 1x106 cells, thus the number of pooled limbs was determined based on the experiment. RNA-seq experiments were performed in duplicates from pools of 2-6 limbs. For H3K27ac ChIP-seq, experiments were performed from singlicates of 2-10 FACS sorted limbs. For C-HiC, experiments were performed from singlicates of 4-12 FACS sorted limbs. These are commonly accepted samples sizes for these experimental techniques. Moreover, each replicate is the result of an average measurement across several pooled samples. For skeletal preparation experiments, 2-4 pairs of limbs were analysed, only one was imaged. Whole-mount in suit hybridization experiments were performed in duplicates, only one was imaged.
Data exclusions	No data was excluded from this study.
Replication	Experiments were performed in replicates when the results were likely to have a significant impact on the result interpretation. FACS-sorted and bulk RNA-seq were performed in duplicates, ChIP-seq and C-HiC were performed in singlicates. Flow cytometry experiments were performed in replicates when FACS based experiments required so, the signal being pooled for graphical representation. Skeletal preparation and Whole-mount in suit hybridization experiments were imaged in singlicates. All attempts at replication were successful.
Randomization	In this work, it was necessary to know the genotypes and types of analyzed cells as they needed to be pooled prior to processing for experiments.
Blinding	Investigators were not blinded since micro-dissection of mouse embryos and further analyses require knowledge about the genotypes and type of cells (EGFP+/-) at hand.

Reporting for specific materials, systems and methods

We require information from authors about some types of materials, experimental systems and methods used in many studies. Here, indicate whether each material, system or method listed is relevant to your study. If you are not sure if a list item applies to your research, read the appropriate section before selecting a response.

Materials & experimental systems

n/a	Involved in the study	
	x	Antibodies
	x	Eukaryotic cell lines
x		Palaeontology and archaeology
	x	Animals and other organisms
x		Clinical data
x		Dual use research of concern
x		Plants

Methods

n/a	Involved in the study	
	x	ChIP-seq
	x	Flow cytometry
x		MRI-based neuroimaging

Antibodies

Antibodies used	Polyclonal rabbit anti-H3K27ac antibody from Diagenode, Cat-No. c15410174, was used at a dilution of 1/500.
Validation	The antibody was validated for ChIP usage on the manufacturer's website: https://www.diagenode.com/en/p/h3k27ac-polyclonal-antibody-classic-50-mg-42-ml. Moreover, we extensively used the same antibody in a prior study and observed a high reproducibility.

Eukaryotic cell lines

Policy information about cell lines and Sex and Gender in Research

Cell line source(s)	Male G4 (129/sv x C57BL/6 F1 hybrid) mouse ESCs (Jorge et al, 2007), male C2 mouse ESCs (Beard et al, 2006) (C57B/6 and 129svJae F1 hybrid) . Both cells lines were obtained from the Nagy laboratory (http://research.lunenfeld.ca/nagy/?page=mouse%20ES%20cells)
Authentication	Genetically modified pluripotent mouse ESCs were authenticated by the production of fetuses through tetraploid aggregations (Artus and Hadjantonakis 2011) and further genotyping confirmed the presence of the desired mutations in the cells.
Mycoplasma contamination	All cell lines tested negative for mycoplasma contamination.
Commonly misidentified lines (See ICLAC register)	No commonly misidentied cell lines were used.

Animals and other research organisms

Policy information about studies involving animals; ARRIVE guidelines recommended for reporting animal research, and Sex and Gender in Research

Laboratory animals	Fetuses were generated from male G4 mouse ESCs (Jorge et al., 2007) or male C2 mouse ESCs (Beard et al., 2006) via tetraploid complementation (Artus and Hadjantonakis 2011). Donor tetraploid embryos were provided from in vitro fertilisation using C57Bl6J x B6D2F1 backgrounds. Aggregated embryos were transferred into CD1 foster females and micro-dissected at embryonic stages E12.5 or E18.5. Three- to twelve-month-old foster females were housed under standard conditions before sacrifice.
Wild animals	No wild animals were used in this study.
Reporting on sex	Fetuses were generated from Male G4 mouse ESCs (Jorge et al, 2007) or male C2 mouse ESCs (Beard et al, 2006).
Field-collected samples	There was no field collected animals in this study.
Ethics oversight	Animal procedures performed in Geneva were in accordance with institutional, state, and government regulations (Canton de Genève authorizations GE/89/19 and GE192A). Animal work performed at Lawrence Berkeley National Laboratory (LBNL) was reviewed and approved by the LBNL Animal Welfare Committee.

Note that full information on the approval of the study protocol must also be provided in the manuscript.

Plants

Seed stocks

N/A

Novel plant genotypes

N/A

Authentication

N/A

ChIP-seq

Data deposition

☒ Confirm that both raw and final processed data have been deposited in a public database such as GEO.

☒ Confirm that you have deposited or provided access to graph files (e.g. BED files) for the called peaks.

Data access links
May remain private before publication.

Datasets are available through the GEO repository under the accession number GSE259212, ChIP-seq data in particular is available under the accession number GSE256337, https://www.ncbi.nlm.nih.gov/geo/query/acc.cgi?acc=GSE256337

Files in database submission

H3K27ac-FL-E125-Inv1-GFPp_macs_SR200_norm.bw
H3K27ac-FL-E125-Rel2-GFPp_macs_SR200_norm.bw
H3K27ac-FL-E125-Rel3-GFPp_macs_SR200_norm.bw
H3K27ac-FL-E125-Inv1-GFPn_macs_SR200_norm.bw
H3K27ac-FL-E125-Inv1-GFPp_macs_SR200_peaks.narrowPeak
H3K27ac-FL-E125-Rel2-GFPp_macs_SR200_peaks.narrowPeak
H3K27ac-FL-E125-Rel3-GFPp_macs_SR200_peaks.narrowPeak
H3K27ac-FL-E125-Inv1-GFPn_macs_SR200_peaks.narrowPeak
H3K27Ac_FL_E125_Inv1_GFPp_S1_L007_R1_001.fastq.gz
H3K27ac-FL-E125-Rel2-GFPp_S13_L001_R1_001.fastq.gz
H3K27ac-FL-E125-Rel3-GFPp_S14_L001_R1_001.fastq.gz
H3K27ac-FL-E125-Inv1-GFPn_S18_L007_R1_001.fastq.gz

Genome browser session
(e.g. UCSC)

N/A

Methodology

Replicates

ChIP-seq were performed in singlicates from FACS-sorted cells obtained from pooled limbs.

Sequencing depth

H3K27Ac_FL_E125_Inv1_GFPp_S1_L007_R1_001.fastq.gz: 64769828
H3K27ac-FL-E125-Rel2-GFPp_S13_L001_R1_001.fastq.gz: 73189768
H3K27ac-FL-E125-Rel3-GFPp_S14_L001_R1_001.fastq.gz: 79952057
H3K27ac-FL-E125-Inv1-GFPn_S18_L007_R1_001.fastq.gz: 28298683

Antibodies

H3K27Ac ChIP-seq was performed using C15410174 (Diagenode) with 1/500 dilution of the antibody.

Peak calling parameters

Peak calling was obtained after extension of the reads by 200 bp using MACS2 v2.2.7.1 (callpeak --nomodel --call-summits --extsize 200).

Data quality

Data quality was manually confirmed by observation by the observation of a strong enrichment of H3K27ac signal at known limb developmental loci, such as the Hox clusters.

Software

TruSeq adapter sequences and bad quality bases were removed using CutAdapt v1.18 (-a AGATCGGAAGAGCACACGTCTGAACTCCAGTCAC -q30 -m15). Reads were processed with the following softwares with default parameters. Reads were then mapped to GRCm39/mm39 using Bowtie2 v2.3.5.1 with default parameters. Reads were then filtered for a MAPQ≥30 using Samtools view v1.10 and the coverage and peak calling was obtained after extension of the reads by 200 bp using MACS2 v2.2.7.1 (callpeak --nomodel --call-summits --extsize 200). Coverage was normalized by the number of million tags used by MACS2.

Flow Cytometry

Plots

Confirm that:

☒ The axis labels state the marker and fluorochrome used (e.g. CD4-FITC).

☒ The axis scales are clearly visible. Include numbers along axes only for bottom left plot of group (a 'group' is an analysis of identical markers).

☒ All plots are contour plots with outliers or pseudocolor plots.

☒ A numerical value for number of cells or percentage (with statistics) is provided.

Methodology

Sample preparation	Sample preparation followed a common protocol for all FACS based experiments and for scRNA-seq. E12.5 limb tissues were microdissected in cold PBS and pooled for processing. To maintain efficiency in downstream experiments, no more than 6 limbs were pooled together at a time. The tissues were dissolved in 400µL Trypsin-EDTA and 40µL 2.5% BSA (Sigma Aldrich, A7906-100G) over 12 minutes at 37°C in a Thermomixer set at 1500 rpm, with a brief resuspension at the 6-minute mark. Trypsin was quenched by adding 400µL 2.5% BSA, and the homogenised tissue was passed through a 40µm cell strainer. An additional volume of 2.5% BSA was passed through to collect any remaining cells. The collected cells were then centrifuged 5' at 4°C and 400 x g, followed by resuspension in 1% BSA. If H3K27ac ChIP was planned as a downstream experiment, 5mM NaButyrate was added to the 1% BSA.
Instrument	BioRad S3
Software	Data was formatted using FlowJo v10.9.
Cell population abundance	Borders to determine population abundance are displayed in main and supplementary figures
Gating strategy	Fluorescence-activated cell sorting (FACS) was employed to identify and sort distinct cell populations in this study, utilizing the Biorad S3 with GFP laser (excitation wavelength 488nm). To eliminate debris from the analysis, FCC/FCS settings were established between 30/40 and 230/220. The viability stain Draq7 was employed to distinguish live cells, and standard protocols were applied to select for singlets.

☒ Tick this box to confirm that a figure exemplifying the gating strategy is provided in the Supplementary Information.
